# Robotically assisted laparoscopic lateral suspension: a step-by-step approach aiming to standardize a novel procedure

**DOI:** 10.1007/s00192-023-05477-6

**Published:** 2023-02-24

**Authors:** Sören Lange, Kyriaki Chatziioannidou, Patrick Dällenbach

**Affiliations:** 1 Department of Gynecology and Obstetrics, Division of Gynecology, Urogynecology Unit, University Hospital Vienna, Vienna, Austria; 2 Medical University Vienna, Waehringer Guertel 18, 1090 Vienna, Austria; 3 Department of Gynecology and Obstetrics, GHOL Group, Nyon Hospital, Nyon, Switzerland; 4 Department of Pediatrics, Gynecology and Obstetrics, Division of Gynecology, Urogynecology Unit, Geneva University Hospitals, Geneva, Switzerland

**Keywords:** Pelvic organ prolapse, Robotic surgery, Laparoscopic lateral suspension, Uterine prolapse

## Abstract

**Introduction and hypothesis:**

The aim of this video is to show a step-by-step approach to robotically assisted laparoscopic lateral suspension for pelvic organ prolapse aiming to standardize this procedure.

**Methods:**

This video shows a robotically assisted laparoscopic approach to a POP-Q stage 3 prolapse with a combined anterior and apical defect. First, the trocars are positioned, with one 8-mm trocar, two lateral trocars 5 cm above the anterior–superior iliac spine, and a 10-mm assistant trocar either paraumbilically or suprapubically. Second, the uterovesical pouch is dissected up to 2 cm above the level of the bladder neck. The mesh is then fixed to the vesicovaginal fascia and to the isthmus uteri. Next, a laparoscopic forceps is inserted retroperitoneally through the lateral trocars and the lateral arms of the mesh are pulled retroperitoneally. The peritoneum of the uterovesical fold is sutured, including round ligament plication. Finally, the lateral arms of the peritoneum are fixed to the peritoneum of the abdominal wall.

**Conclusions:**

Robotically assisted laparoscopic lateral suspension is a safe alternative to laparoscopic and robotically assisted laparoscopic sacropexy and very well suited for uterine-preserving POP surgery. This video contributes to the standardization of this procedure, and we believe our video to be useful in helping urogynecologists to perform this innovative procedure.

**Supplementary information:**

The online version contains supplementary material available at 10.1007/s00192-023-05477-6

## Introduction

Laparoscopic hysteropexy by lateral suspension (LLS) with mesh is a technique to repair symptomatic anterior and apical pelvic organ prolapse (POP) [1]. It is considered an alternative to minimal invasive sacrohysteropexy. Robotic assistance helps to avoid the lateral transparietal passage of the laparoscopic technique thereby avoiding potential damage to the ilioinguinal and iliohypogastric nerves while reducing the number of scars [2, 3]. Observational studies showed high objective cure rates of 83–94% and high subjective cure rates of 77–96% [3, 4]. Reoperation rates for recurrence were between 5 and 9%, a range similar to reoperation rates after sacropexy. Additionally, to the above-mentioned advantages of robotically assisted lateral suspension (RALLS), we also advocate for a temporary fixation of the lateral arms of the mesh with i.e., resorbable staplers to lower the risk of mesh arm displacement until fibrosis suspends the arms without tension, a technique we use since 2010 [3, 5]. While in some studies the laparoscopic technique described by our team in 2011 was similarly used for robotically assisted procedures, we advocate to benefit from the robotical assistance to the advantage of the patient [3, 4, 6]. To achieve this, trocar placement during RALLS has to be different from LLS [7]. The impact of these modifications on patient’s outcome is unknown, given that no comparative trials were conducted. Prior to more impactful modifications, LLS should be compared in randomized controlled trials against the current standard, sacropexy, and ideally, LLS should be standardized to permit repeatability of these trials.

We lack standardization for even the most frequently performed prolapse surgeries [8, 9]. To guarantee the production of impactful research data and patients’ safety, standardization of terminology and techniques is important [10]. The WHO has implemented standardization practices to achieve consistency in procedures and to reduce failure rates [11]. Aiming at a standardization of this procedure, we propose a step-by-step approach to robotically assisted laparoscopic lateral suspension (RALLS) to be implemented in future clinical trials.

## Materials and methods

This video shows a robotically assisted laparoscopic approach to a symptomatic POP-Q stage 3 prolapse with a combined anterior and apical defect (Aa +3, Ba +4, C +1, gh 4, pb 4, tvl 8, Ap −3, Bp −3, D −6) in a 73-year-old patient. After a thorough discussion about therapeutic options, the patient opted for a surgical repair by robotic hysteropexy with lateral suspension. The procedure was performed under general anesthesia.

In our institution we use the da Vinci system by Intuitive Surgical®. All patients receive preoperative prophylactic antibiotics (mefoxitin 2 g intravenously, or clindamycin 600 mg in the case of allergy) at induction of anesthesia. We use a titanized macroporous polypropylene mesh (TiLOOP® “Prof Dubuisson”® 9X 41.5 cm, 65 g/m^2^). At the beginning of the procedure the patient is put in the dorsal lithotomy position. After visualization of the pelvic organ prolapse, we place a uterine manipulator and an indwelling catheter. This helps to provide sufficient organ exposure during the intervention and reduces the risk of organ damage. The ureters are always controlled before starting the dissection. Additionally, an intravaginal retractor manipulated by an assistant sitting between the legs of the patient helps to visualize the uterovesical fold.

**Fig. 1:**
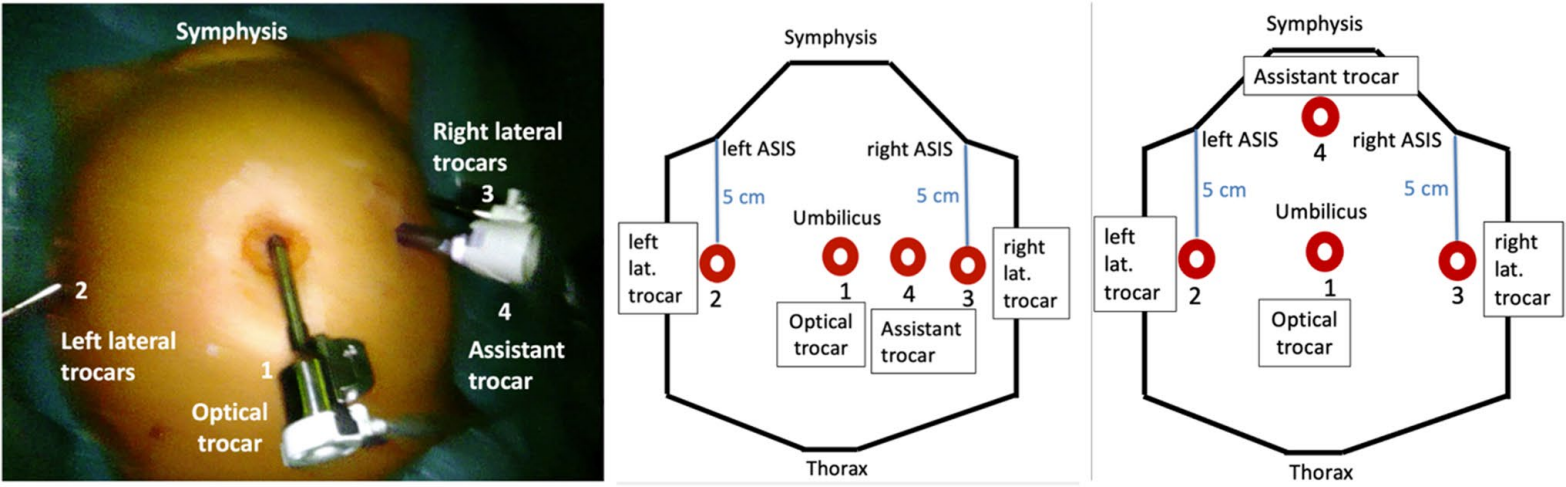
Positioning of the trocars. *Left*: intraoperative view. *Center*: schematic representation with positioning of the assistant trocar between the optical and the right lateral trocar (in the case of Da Vinci models S, Si, X). *Right*: schematic representation with positioning of the assistant trocar suprapubically (in the case of Da Vinci model Xi). *1*, optical trocar. *2* and *3*, lateral trocars. *4*, assistant trocar. *ASIS* anterior superior iliac spine

**Fig. 2:**
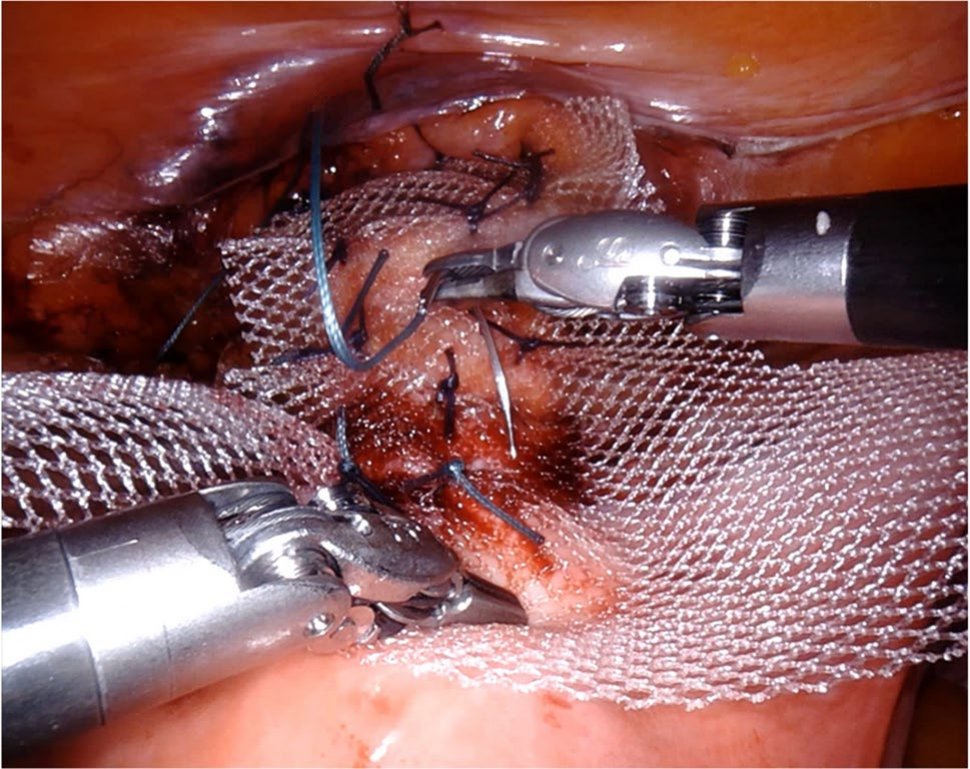
Fixation of the mesh to the vesicovaginal fascia and to the isthmus uteri

**Fig. 3:**
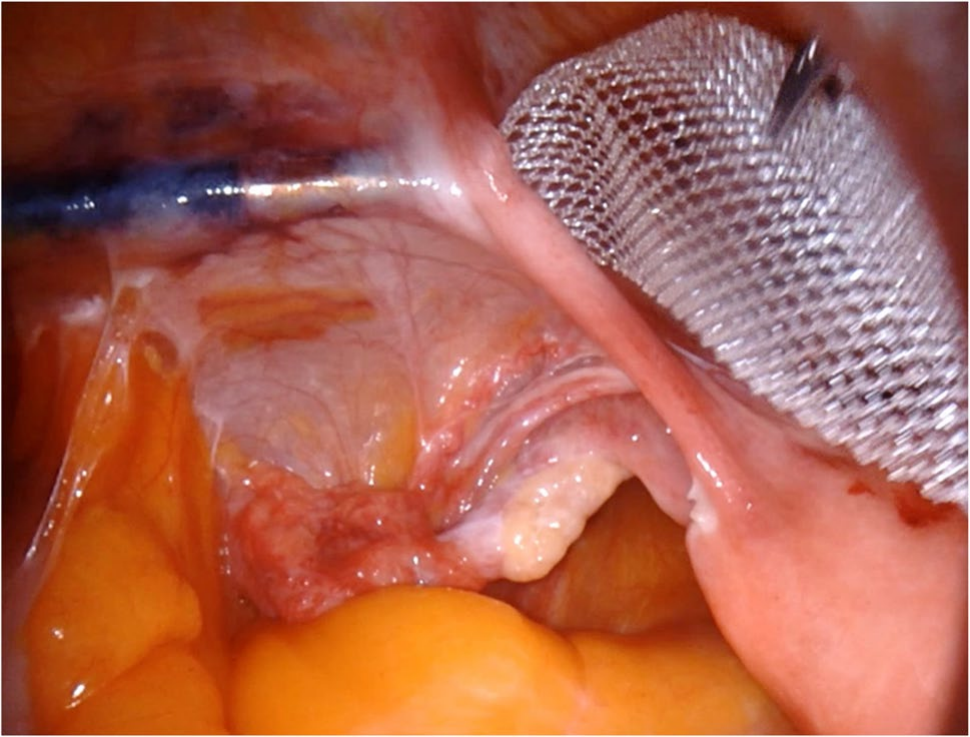
Passage of the lateral branch of the mesh under the round ligament on the left-hand side

1. Positioning of the trocars: we use an 8-mm umbilical trocar, and two lateral 8-mm trocars, placed laterally 5 cm above the anterior–superior iliac spine (ASIS). The lateral placement of the trocars above the ASIS allows the assistant to pull up the lateral arms of the mesh through the trocars, thus avoiding supplementary incisions and a transparietal passage of the lateral arms of the mesh, as is performed during the laparoscopic technique. Robotic assistance made the very lateral positioning of the trocars feasible, whereas this placement would be ergonomically problematic in laparoscopic lateral suspension. A fourth, 10-mm assistant trocar is put either paraumbilically (for Da Vinci models S, SI, X) or nowadays most often suprapubically (for Da Vinci model Xi see Fig. 1).
2. Dissection of the uterovesical pouch up to 2 cm above the level of the bladder neck: during this step, the assistant, who is positioned between the legs of the patient, helps to identify the bladder neck by putting two fingers into the patient’s vagina. Additionally, mobilization of the balloon of the indwelling catheter by the assistant might help to identify the caudal limits of dissection.
3. Fixation of the mesh to the vesicovaginal fascia with nonpermanent sutures and to the isthmus uteri with permanent sutures (Fig. 2): the mesh is sutured to the vesicovaginal fascia using nonpermanent sutures of 2–0 polyglactin 910 (Vicryl™ 2–0, JB needle, Ethicon). The fixation to the isthmus uteri is performed with permanent polyester 0 sutures (Ethibon Excel™ 0, CT-1 needle, Ethicon).
4. Retroperitoneal insertion of a laparoscopic forceps through the lateral trocars: after retraction of the lateral robotic instruments, the assistant inserts a laparoscopic forceps via the lateral trocars on each side. Care should be taken not to perforate the peritoneum and to always visualize the grasping forceps through the transparent peritoneum. The movement should be directed caudally and slightly anteriorly, aiming at the middle portion of the round ligament.
5. Retroperitoneal pulling of the lateral arms of the mesh: this step is performed by the field assistant, who takes care not to injure the external iliac blood vessels (Fig. 3). When the lateral mesh arms are pulled in place, the laparoscopic forceps is retracted, and the lateral robotic instruments are reinserted.
6. Suture of the peritoneum of the uterovesical fold including plication of the round ligaments: A simple overlock suture of uninterrupted polyglactin 910 (Vicryl™ 0, CT-2 needle, Ethicon) is performed.
7. Fixation of the lateral arms of the mesh to the peritoneum of the abdominal wall: first, we check that there is no excessive tension on the mesh. Then, the field assistant fixes the arms of the mesh to the peritoneum of the abdominal wall with absorbable tacks (AbsorbaTack® fixation device, Covidien).

Patients receive postoperative fractional heparin. A vaginal swab with estrogen cream (Oestro-Gynaedron®) is placed in the vagina over night.

The 1-year check-up showed a POP-Q score of Aa −3, Ba −3, C −7, gh 3, pb 4, tvl 8, Ap −1, Bo −1, and D −8.

## Conclusion

Robotically assisted laparoscopic lateral suspension (RALLS) with mesh is a safe alternative to laparoscopic and robotically assisted laparoscopic sacropexy and very well suited to uterine-preserving POP surgery [3, 4]. Nevertheless, this novel procedure lacks standardization. Standardization of procedures is needed to reduce failure rates, to produce impactful research data, and to improve patients’ safety [10, 11]. This video contributes to the standardization of this procedure, and we believe our video to be useful in helping urogynecologists to perform this innovative procedure.

## Supplementary information

Below is the link to the electronic supplementary material.

Supplementary file1 (MP4 104083 KB)

## Data Availability

Data sharing not applicable to this article as no datasets were generated or analysed during the current study.

## Abbreviations

ASIS: Anterior superior iliac spine
LLS: Laparoscopic lateral suspension
POP: Pelvic organ prolapse
POP-Q: Pelvic organ prolapse quantification
RALLS: Robotically assisted laparoscopic lateral suspension
WHO: World Health Organization

## References

[1] Veit-Rubin N, Dubuisson J, Constantin F (2019). Uterus preservation is superior to hysterectomy when performing laparoscopic lateral suspension with mesh. Int Urogynecol J.

[2] Dällenbach P, Veit N (2014). Robotically assisted laparoscopic repair of anterior vaginal wall and uterine prolapse by lateral suspension with mesh: initial experience and video. Int Urogynecol J.

[3] Dällenbach P, Alec M, Boulvain M, Shabanov S (2021). Outcomes of robotically assisted laparoscopic lateral suspension (RALLS) with mesh for anterior and apical prolapse. J Robot Surg.

[4] Russo E, Giannini A, Guevara MM, Mannella P, Misasi G, Falcone M, et al. Medium-term outcomes after robotic-assisted lateral suspension with mesh for advanced multi-compartmental prolapse. Int Urogynecol J. 2020;31(8):1647–53. 10.1007/s00192-019-04069-7 PMC7363728 31388718

[5] Chatziioannidou K, Veit-Rubin N, Dällenbach P. Laparoscopic lateral suspension for anterior and apical prolapse: a prospective cohort with standardized technique. Int Urogynecology J. 2022;33(2):319–25. 10.1007/s00192-021-04784-0 PMC8803665 33835212

[6] Dubuisson JB, Eperon I, Jacob S, Dubuisson J, Wenger JM, Dallenbach P, Kaelin-Gambirasio I. Laparoscopic repair of pelvic organ prolapse by lateral suspension with mesh: a continuous series of 218 patients. Gynecol Obstet Fertil. 2011;39(3):127–31. 10.1016/j.gyobfe.2010.12.007 21377391

[7] Simoncini T, Russo E, Mannella P, Giannini A (2016). Robotic-assisted apical lateral suspension for advanced pelvic organ prolapse: surgical technique and perioperative outcomes. Surg Endosc.

[8] Halpern-Elenskaia K, Umek W, Bodner-Adler B, Hanzal E (2018). Anterior colporrhaphy: a standard operation? Systematic review of the technical aspects of a common procedure in randomized controlled trials. Int Urogynecol J.

[9] O’Sullivan OE, Matthews CA, O’Reilly BA (2016). Sacrocolpopexy: is there a consistent surgical technique?. Int Urogynecol J.

[10] Developed by the Joint Writing Group of the American Urogynecologic Society and the International Urogynecological Association (2020). Joint report on terminology for surgical procedures to treat pelvic organ prolapse. Int Urogynecol J.

[11] Leotsakos A, Zheng H, Croteau R (2014). Standardization in patient safety: the WHO high 5s project. Int J Qual Health Care.

